# Randomized trials of artemisinin-piperaquine, dihydroartemisinin-piperaquine phosphate and artemether-lumefantrine for the treatment of multi-drug resistant falciparum malaria in Cambodia-Thailand border area

**DOI:** 10.1186/1475-2875-10-231

**Published:** 2011-08-10

**Authors:** Jianping Song, Duong Socheat, Bo Tan, Suon Seila, Ying Xu, Fengzhen Ou, Sreng Sokunthea, Leap Sophorn, Chongjun Zhou, Changsheng Deng, Qi Wang, Guoqiao Li

**Affiliations:** 1 Research Center for Qinghao (Artemisia annual L.), Guangzhou University of Chinese Medicine, Guangzhou, China; 2 National Centre for Parasitology, Entomology and Malaria Control, Phnom Penh, Cambodia; 3 Pursat Referral Hospital, Pursat, Cambodia

## Abstract

**Background:**

Drug resistance of falciparum malaria is a global problem. Sulphadoxine/pyrimethamine-resistant and mefloquine-resistant strains of falciparum malaria have spread in Southeast Asia at lightning speed in 1980s-1990s, and the Cambodia-Thailand border is one of the malaria epidemic areas with the most severe forms of multi-drug resistant falciparum malaria.

**Methods:**

Artemisinin-piperaquine (AP), dihydroartemisinin-piperaquine phosphate (DHP) and artemether-lumefantrine (AL) were used to treat 110, 55 and 55 uncomplicated malaria patients, respectively. The total dosage for adults is 1,750 mg (four tablets, twice over 24 hours) of AP, 2,880 mg (eight tablets, four times over two days) of DHP, and 3,360 mg (24 tablets, six times over three days) of AL. The 28-day cure rate, parasite clearance time, fever clearance time, and drug tolerance of patients to the three drugs were compared. All of the above methods were consistent with the current national guidelines.

**Results:**

The mean parasite clearance time was similar in all three groups (66.7 ± 21.9 hrs, 65.6 ± 27.3 hrs, 65.3 ± 22.5 hrs in AP, DHP and AL groups, respectively), and there was no remarkable difference between them; the fever clearance time was also similar (31.6 ± 17.7 hrs, 34.6 ± 21.8 hrs and 36.9 ± 15.4 hrs, respectively). After following up for 28-days, the cure rate was 95.1%(97/102), 98.2%(54/55) and 82.4%(42/51); and the recrudescence cases was 4.9%(5/102), 1.8%(1/55) and 17.6%(9/51), respectively. Therefore, the statistical data showed that 28-day cure rate in AP and DHP groups was superior to AL group obviously.

The patients had good tolerance to all the three drugs, and some side effects (anoxia, nausea, vomiting, headache and dizziness) could be found in every group and they were self-limited; patients in control groups also had good tolerance to DHP and AL, there was no remarkable difference in the three groups.

**Conclusions:**

AP, DHP and AL all remained efficacious treatments for the treatment of falciparum malaria in Cambodia-Thailand border area. However, in this particular setting, the AP regimen turned out to be favourable in terms of efficacy and effectiveness, simplicity of administration, cost and compliance.

**Trial Registration:**

The trial was registered at *Chinese Clinical Trial Register *under identifier 2005L01041.

## Background

Malaria kills around one million people each year, although is a preventable and treatable infectious disease [1]. Because malaria is a disease that affects mostly poor and vulnerable population, it perpetuates a vicious cycle of poverty in the developing world. Malaria can be prevented, diagnosed and treated with a combination of available tools. However, it has been estimated that USD 4.2 billion is needed each year to fully fund the fight against malaria [2].

Drug resistant falciparum malaria is a global problem. Sulphadoxine/pyrimethamine and mefloquine resistant strains of falciparum parasites have spread quickly throughout Southeast Asia in 1980s-1990s, and the Cambodia-Thailand border is one of the endemic regions with the most severe multi-drug resistant falciparum malaria [3-6]. WHO has recommended the use of artemisinin combination therapy (ACT) to improve anti-malarial effectiveness and to keep the selection of drug-resistant parasites to a minimum [7]. Now more than 60 countries have switched to ACT for treating malaria, however, the wide-spread application of ACT has been limited by high cost [8,9].

Dihydroartemisinin-piperaquine phosphate in a fixed-dose preparation of dihydroartemisinin 40 mg and piperaquine phosphate 320 mg has been shown to be highly effective in treating falciparum malaria with an efficacy of over 90% in China, Vietnam, and Cambodia [10-12]. Artemether-lumefantrine (20 mg of artemether and 120 mg of lumefantrine) is another ACT that is administered in six consecutive doses: four tablets each at 0 hours and 8 hours on the first day, then twice daily on the two consecutive days. Lumefantrine is a highly lipophilic substance, and the oral bioavailability varies considerably among individuals, and increases greatly if the drug is administered after a meal rich in fat [13]. This remains a limitation to the effective usage of the drug because uptake may be reduced in fasting patients.

Artemisinin-piperaquine was recently developed by Chinese scientists and has shown good efficacy and safety in a two-day treatment course [14]. Piperaquine is a 4-chloroquinoline (1,3-bis[1-(7-chloro-4'-quinolyl)-4'-piperazinyl]) and was used to replace chloroquine in China in 1970s-1980s. It has been shown that patients' tolerance to piperaquine was better than to piperaquine phosphate. Additionally, piperaquine can reduce the treatment time and cost [9,15].

The pharmacodynamics studies of artemisinin and its derivatives have shown that artemisinin (ART) and its derivatives are characterized by a quick absorption, wide distribution and quick excretion *in vivo *[16]. Clinical trials of ART in the treatment of uncomplicated falciparum malaria, conducted in 2002 (Li Guoqiao and others, unpublished data) showed that ART could quickly inhibit the growth of parasite at the dosage of 2 mg/kg with no significant difference in the parasite clearance time betweem ART and dihydroartemisinin (DHA) at the same dosage. DHA is the active metabolite of artemether and artesunate, which are widely used, and ART derivative must be given for 7 days for optimum cure rate if administered alone. Therefore, combining an ART derivative with another anti-malarial drug with a long half-life will shorten treatment time and is considered the treatment of choice for falciparum malaria [8,17].

In this trial, the effectiveness and safety of the three drugs for the treatment of uncomplicated falciparum malaria were compared and evaluated for developing an optimal anti-malarial regimen.

## Methods

### Study site and recruitment procedures

The trial was conducted in Pursat Referral Hospital, Pursat province, Cambodia. Pursat is located in the western part of the country bordering Thailand. It is located between the Tonle Sap lake and the northern end of the Cardamom Mountains. The patients for this trial came from the forest area in the Cambodia and Thailand boarder, where multi-drug resistant falciparum malaria is widespread.

The implementation of this study is according to the "Agreement of Cambodia-China Fast Malaria Control Co-operation" and "National Ethics Committee for Health Research (No.03G/03NECHR)" signed by Guangzhou University of Chinese Medicine, China, and the Ministry of Health, Kingdom of Cambodia.

### Enrolment

It was a prospective, open labeled, randomized controlled trial conducted on patients with microscopically confirmed symptomatic of falciparum malaria, between seven and 65 years of age. All patients with symptoms of malaria, such as fever, and who had not been administrated any anti-malarial drug, including sulphonamide, tetracycline or trimethoprim, in the previous week, were enrolled [18]. A standard data sheet was used to record demographic information, details of symptoms and their duration, and anti-malarial history. Clinical examination, vital signs and axillary temperature were documented. Giemsa-stained thick and thin blood films were used to confirm parasite species and parasitaemia.

Inclusion criteria: (1) patients with clinical symptoms of malaria, such as fever (axillary temperature≥37.5°C), (2) age ranged from seven to 65 years, (3) peripheral blood smears showed that falciparum malaria asexual parasite were 1,000~100,000/μl, (4) no anti-malarial had been taken within seven days prior to the onset of symptoms or admission, including sulphonamide, tetracycline or trimethoprim.

Exclusion criteria: (1) pregnant women;(2) lactating mothers; (3) children under seven years of age, senior persons elder than 65 years of age; (4) patients with severe vomiting or diarrhoea, or other signs of severe malaria; (5) febrile diseases other than falciparum malaria; (6) exiting before the 28-day follow up and/or lack of follow-up data; (7) patients identified as re-infection by PCR were also excluded from the final cure rate and recrudescence rate calculation.

### Randomization and masking

Patients were allocated to three treatment arms based on a pre-generated randomization list made in blocks of 20 that was produced and held independently of the field teams by a statistician. The individual allocations were kept in sealed, opaque envelopes and opened only after enrolment by the field team. Patients and clinical field workers were not blinded to the treatment arm after allocation. Microscopists were blinded to treatment allocation at follow-up examinations. Adverse events were assessed by symptom enquiry at every visit.

### Drugs and regimen

AP tablets containing 62.5 mg artemisinin (ART) and 375 mg piperaquine (PQ) per tablet were provided by Artepharm Co., Ltd., China (batch No. was 20030820). DHP tablets containing 40 mg dihydroartemisinin (DHA) and 375 mg piperaquine phosphate (PQP) were provided by Guangzhou Holleykin Pharmaceutical Co., Ltd. (batch No. was 20030301). AL tablets containing 20 mg artemether and 120 mg lumefantrine were obtained from Beijng Novartis Pharm Ltd. (batch No. was 20030822). The regimens of AP, DHP and AL were listed in Additional file 1, Table S1.

### Follow-up

At enrollment, a full physical examination, including a neurological examination and a medical history, was performed. Thick and thin blood smears were examined by microscopy, and routine haematology was performed. Drug administration was observed by study physicians. If vomiting occurred within 1 h of dosing, the medications were re-administrated. The patients were kept in the hospital for 7 days or until parasites were cleared and were followed-up weekly till D28 from the start of treatment or whenever he/she felt sick within 28 days. At each follow-up, blood smear was done via a finger prick. Blood spots were collected from those with reappearance of asexual parasitaemia. All information was recorded on a standard case record form.

### Sample size

A total of 245 cases of uncomplicated malaria were enrolled for this study. The patients were separated into three random groups with 122 cases in AP group, 61 cases in DHP group and 62 cases in AL groups. And complete data from 110, 55 and 55 patients, respectively, were obtained and analyzed.

### Laboratory tests and outcome measurement

Patients were observed in the hospital for 7 days and followed up at D14, D21 and D28 for any symptoms or malaria parasites changes. Temperature was taken every six hours and once daily after it returned to normal(37.5°C) for 24 hours. Ward rounds were done twice daily in the morning and afternoon, and side effects were inquired and recorded according to the "Side Effects Record Form".

Thick and thin blood smears were taken at 7 AM and 5 PM from D0 to D4 to calculate the number of asexual form per 200 WBCs (White Blood Cells). If the number of parasites < 1/200 WBC, 200 fields were examined to identify parasite clearance. Three consecutive negative blood smears (from D5 to D7) indicated clearance of parasite. For follow-ups, blood smears were taken at D14, D21 and D28 to investigate recrudescence. If a patient had fever, he or she was advised to go back to the hospital for further examination. Blood spots collected D0 and positive blood smears during 28-day follow up were kept for qualitative PCR analysis. Blood spots of patients whose blood smears were positive within 28-day follow up were sent to the Oxford University Clinical Research Unit, Hospital for Tropical Diseases, Ho Chi Minh City, Vietnam, for PCR qualitative analysis to differentiate re-infection and recrudescence.

WBC and RBC (Red Blood Cell) counts and electrocardiogram examination were done at D0 and D7. Alanine aminotransferase (ALT), aspartate aminotransferase (AST), total bilirubin (TBIL), direct bilirubin (DBIL), creatinine (CR) and blood urea nitrogen(BUN)were also examined at D0 and D7. Abnormal items found at D7 were re-examined every week until the patient recovered.

The following four main efficacy parameters were used: Early Treatment Failure (ETF); Late Clinical Failure (LCF); Late Parasitological Failure (LPF); Adequate Clinical and Parasitological Response (ACPR) [18]. The occurrence rate of side effects (any symptoms and objective sign having appeared or aggravated after the administration) was recorded. Functions of liver and kidney and electrocardiogram before and after drug administration were checked in order to evaluate the toxicity and side effects of drug to the major organs.

### Polymerase chain reaction (PCR)

The patients in the trial continued to live in the area of transmission, therefore, re-infection during the four weeks of follow-up was possible. Then, PCR-genotyping were used to distinguish recrudescence from re-infection. Blood samples were taken at baseline (D0) and on the day of reappearance of parasitaemia [1]. The nested PCR of three polymorphic marker genes [Merozoite Surface Antigen-1 (MSA-1), Merozoite Surface Antigen-1 (MSA-2), and Glutamine-rich Protein (GLURP)] were conducted in University of Oxford Clinical Research Unit, Hospital for Tropical Diseases, Ho Chi Minh City, Vietnam. PCR products were analyzed by agarose gel electrophoresis. Patients with the same parasite PCR profiles in the first and second infections were considered to have a recrudescent infection.

### Statistical analysis

Statistic analysis was conducted using SAS 6.12. Data were presented as means ± standard deviation (SD) or as the geometric mean and range. All statistical tests were two-tailed, and a significance level of 0.05 was used.

### Ethical approval

The study was approved by the National Ethics Committee for Health Research of the Ministry of Health, Phnom Penh, Kingdom of Cambodia, and the Ethics Committee of Guangzhou University of Chinese Medicine, Guangzhou, China. This ethics approval of the trial describes recruitment of patients with uncomplicated infection with *P. falciparum*, with treatments for AP, DHP and AL, then research on the therapeutic efficacy and safety. The clinical trials registry number was 2005L01041.

## Results

### Patient recruitment and participant flow

**Figure 1:**
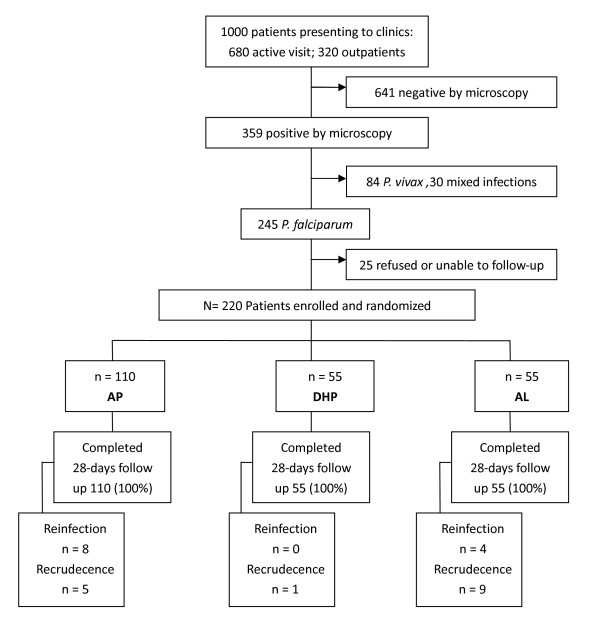
**Patients flow chat in the clinical trial**.

From 22 July, 2005 to 18 October, 2005, 220 patients completed in the study with 110 receiving AP, 55 receiving DHP and 55 receiving AL (Figure 1). Common reasons for exclusion included lack of follow-up data, and patients exiting before the 28-day follow up. Among the 220 patients completing the clinical trial, 18 (8.18%) were 7-10 years of age, 26 (11.82%) were 11-15 years of age, and 176 (80.00%) were more than 15 years old. A flow chart of patient enrollment procedures and treatment is shown in Figure 1. Baseline characteristics of the recruited patients are similar, and it is shown in Table 1.

**Table 1:** Patient age, symptoms, and other clinical measurements

Characteristic	Treatment arm			
**Number of patients**	**AP** **(n = 110)**	**DHP** **(n = 55)**	**AL** **(n = 55)**	**P** **value**

Gender: Male/Female(ratio)	72/28(2.57)	43/12(3.58)	46/9(5.11)	NS^a^
Mean age, years(range)	25.2(7-55)	21.9(8-64)	27.4(7-58)	NS
Age Groups:				
7-10	11(10.0%)	2(3.6%)	5(9.1%)	NS
11-15	16(14.54%)	5(9.1%)	5(9.1%)	NS
> 15	83(75.46%)	48(87.3%)	45(81.8%)	NS
mean duration of fever (in days) (range)	4.2 (1.3-7.1)	4.6 (1.3-7.9)	5.3 (1.1-9.5)	NS
Mean parasitaemia (1 ×/μl)(range)	26637(3801-49473)	25850(195-51505)	31297(1215-61379)	NS
Body temperature (°C), mean (range)	38.5(37.7-39.3)	38.8 (37.7-39.9)	38.8(37.7-39.9)	NS
Hepatomegly rate (%)	5.5	14.5	7.3	NS
Splenomegly rate (%)	26.4	21.8	12.7	NS

NS: Not significant, P > 0.05

### Treatment effectiveness

There was no early treatment failure in the first three day following initiation of therapy for all the patients with 28-day follow up data. The Adequate Clinical and Parasitological Response (ACPR) of the three groups on D28 were 97/110 (88.2%), 54/55 (98.2) and 42/55 (76.4%), respectively (Table 2). During the 28-day follow up, blood smears of 13 cases were found positive in the AP group. According to the results of PCR qualitative analysis, eight cases were classified as re-infection and five cases were recrudescence (one case recrudesced at D21 and four cases recrudesced at D28). In the DHP group, one blood smear was found positive at D28, and it was identified as recrudescence according to the qualitative PCR analysis. Among the 13 blood smear positive cases in the AL group, four cases were considered re-infection and nine cases were recrudescence (one case recrudesced at D14, three cases at D21 and five cases at D28).

**Table 2:** Efficacy and parasite clearance time of the three treatment groups

Therapeutic response	AP (n = 110)	DHP (n = 55)	AL (n = 55)
Excluded(%)	0	0	0
Lost of follow-up (%)	0	0	0
Early Treatment Failure(%)	0	0	0
Late Clinical Failure(%, 95%CI)	13(11.8, 5.79~17.85%)	1(1.8, -1.71~5.34%)	13(23.6,12.41~34.86%)
ACPR (%, 95% CI) on D14 on D21 on D28	109 (99.1, 97.31~100.86%) 105(95.5, 91.56%~99.35%) 97(88.2, 82.15%~94.21%)	55(100, 93.6%~100%) 55(100, 93.6%~100%) 54(98.2, 94.65~101.71%)	54(98.2, 94.65~101.71%) 48(87.3, 78.46%~96.08%) 42(76.4, 65.14%~87.59%)
ACPR by Intention-to-treat analysis on D28 (%)	97/110 (88.2)	54/55 (98.2)	42/55 (76.4)
ACPR adjusted by PCR on D28 (%)	97/102*^▲^ (95.1)	54/55 (98.2)	42/51 (82.4)
Parasite clearance time, mean ± SD hours	66.7 ± 21.9*^Δ^	65.6 ± 27.3^Δ^	65.3 ± 22.5
Fever clearance time, mean ± SD hours	31.6 ± 17.7*^Δ^	34.6 ± 21.8	36.9 ± 15.4

Note: *P > 0.05, compared with DHP;^▲^P < 0.05, compared with AL;^Δ^P > 0.05, compared with AL.

The 28-day cure rates adjusted after PCR analysis were 97/102 (95.1%), 54/55(98.2%), 42/51 (82.4%) for AP, DHP and AL, respectively (Table 2). There was no significant difference between AP and DHP (P > 0.05). The 28-day cure rate in the AP group was significantly higher than that in the AL group (P < 0.05) (Table 2). The parasite and fever clearance time in the three groups were similar (P > 0.05) (Table 2).

### Side effects

There were no serious adverse events recorded during the trial. All of the three study drugs appeared to be well tolerated.

### Haematology, electrocardiogram and biochemistry

No abnormal changes in haemotology and electrocardiogram were observed in the three study groups. Slightly increases in AST, ALT, BIL, BUN and CR measurements were found at D0 in all three groups, and most of them returned to normal ranges at D7. At D7, there were two cases with high TBIL in the AP group, one case with high AST and two cases with high CR, but they became normal at D14. There were four cases with high CR in the DHP group at D7; again they became normal at D14. There were two cases with high TBIL, three cases with high AST and one case with high BUN in the AL group at D7, and they returned normal at D14.

## Discussion

**Figure 2:**
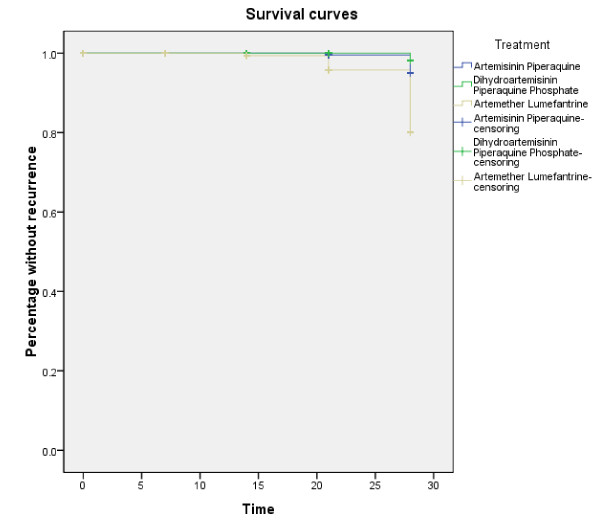
**Survival curves**. (A new Kaplan Meier curve was added.). Recrudescence rate in each group was recorded on D0, D7, D14, D21, and D28, respectively, and further confirmed by PCR qualitative analysis.

This randomized clinical trial showed that both AP and DHP were highly efficacious in clearing parasitaemia and were well tolerated for treatment of uncomplicated malaria in the Cambodia-Thailand border. Lower cure rate was obtained from AL treatment (Figure 2), which was similar to the results reported previously (71.1% cure rate in 2002 and 86.5% in 2003) [19]. The reason may be that, the lack of fat in the patient diet affected the absorption of lumefantrine, one of the partner drugs in AL. Dietary fat had been shown to enhance the bioavailability of artemether and lumefantrine, although this effect was more apparent for lumefantrine [13,20,21]. Administration of AL to healthy volunteers at the same time as a high-fat meal increases the bioavailability of artemether and lumefantrine by two-fold and 16-fold, respectively, compared with the fasting state [13,21].

No obvious side effects related to the drugs were found in all the three groups. Although symptoms of anoxia, nausea, vomiting, headache and dizziness were observed in some patients, which was similar to those observed in Chinese healthy volunteers and in Vietnamese patients, as well as in Rwandan children [10,22-26]. The occurrence rate of side effects was low. Comparing the results of haematology, biochemistry and electrocardiogram between those before and after drug administration showed no obvious toxic reactions related to drugs. The patients in the three groups had good tolerance to the drugs. Although more side effects caused by piperaquine phosphate in DHP than that by piperaquine base in AP combination were reported previously [27,28], the data presented here did not show any differences in the two groups. In 1998, WHO launched Roll Back Malaria campaign, which aimed to reduce the global number of deaths due to malaria in half by 2010 [7]. However, until 2004, the global number of people having died of malaria had not declined, because the high prices of ACT have delayed timely drug distribution [16,18]. In East Africa, failure rates of chloroquine and sulphadoxine-pyrimethamine treatment were 64% and 45%, respectively, and children deaths due to malaria increased by 11-fold [8,29]. Therefore, it is imminent to develop affordable ACT, that is cheaper for people in malaria-endemic countries.

The 28-day cure rate of AP for uncomplicated falciparum malaria was high, the patients had good tolerance to AP as shown above, and by taking four tablets as the total dosage for adults over a 24-hour treatment was convenient and help to improve patients' compliance. Hence, this dosage was recommended for the expanding clinical trial (Phase III).

## Conclusions

In this study, three different forms of ACTs (AP, DHP and AL) were compared in the multi-drug resistant falciparum malaria epidemic areas in the Cambodia-Thailand border.

The data from this report demonstrated that the AP regimen turned out to be favourable in terms of efficacy and effectiveness, simplicity of administration, cost and compliance in this particular setting, and it might play an important role for malaria control in the near future.

## Competing interests

The authors declare that they have no competing interests.

## Authors' contributions

JS organized the program and drafted the manuscript; SD participated in and supervised the program; BT participated in the projects and did the field work. S Seila did the field work and was in charge of patients inclusion; YX did the field work and the clinical observation; S Sokunthea and FO checked the blood smear and did the biochemistry analysis; CZ and QW collected the data; CD collected and analyzed the cases; and GL participated in the design of this study and coordination and helped to draft the manuscript. All authors read and approved the final manuscript.

## Supplementary Material

Additional file 1 **Table S1 - Dosing schedules for artemisinin-piperaquine (AP), dihydroartemisinin-piperaquine phosphate (DHP) and artemether-lumefantrine (AL)**. *It is better to take this drug together with milk and food. Click here for file

## References

[B1] World Health Organization, 2010 World malaria report Geneva: World malaria report (WHO/HTM/GMP/2010.12)

[B2] Roll Back Malaria Partnership: What is malaria? http://www.rollbackmalaria.org/malariaMessages.html

[B3] D'Alessandro U Buttiens H History and importance of antimalarial drug resistance Trop Med Int Health 2001 6 845 848 10.1046/j.1365-3156.2001.00819.x 11703837

[B4] Nosten F Ter Kuile F Chongsuphajaisiddhi T Luxemburger C Webster HK Edstein M Phaipun L Thew KL White NJ Mefloquine-resistant falciparum malaria on the Thai-Burmese border Lancet 1991 337 1140 1143 10.1016/0140-6736(91)92798-7 1674024

[B5] Ter Kuile FO Nosten F Thieren M Luxemburger C Edstein MD Chongsuphajaisiddhi T Phaipun L Webster HK White NJ High-dose mefloquine in the treatment of mutidrugs-resistance falciparum malaria J Infect Dis 1992 166 1393 1400 10.1093/infdis/166.6.1393 1431257

[B6] National Center for Parasitology Entomology and Malaria Control Ministry of Health Annual National Conference of National Center for Parasitology Entomology and Malaria Control 2003 Phnom Penh, Cambodia

[B7] Roll Back malaria Technical Consultation Antimalarial drug combination therapy Geneva: World Health Organization, WHO/CDS/RBM/2001.35

[B8] World Health Organization Guidelines for the treatment of malaria 2006 Geneva: WHO 21 22

[B9] Davis TM Hung TY Sim IK Karunajeewa HA Ilett KF Piperaquine: a resurgent antimalarial drug Drugs 2005 65 75 87 10.2165/00003495-200565010-00004 15610051

[B10] Nguyen XT Trieu NT Nguyen CP Nguyen XT Bui D G Dennis S Marina C Michael DE Open label randomized comparison of dihydroartemisinin-piperaquine and artesunate-amodiaquine for the treatment of uncomplicated *Plasmodium falciparum *malaria in central Vietnam Trop Med Int Health 2009 14 504 511 10.1111/j.1365-3156.2009.02269.x 19320869

[B11] Song JP Duong S Suou S Thou T Ses S Sim Y Tan B Li GQ Clinical research of dihydroartemisinin-piperaquine with uncomplicated falciparum malaria Natl Med J China 2003 83 1099 1100

[B12] Mey BD Timothy MED Sean H Sandra I Khim N Thierry F Yi P Chiv L Doung S Efficacy and safety of dihydroartemisinin-piperaquine (DHP) in Cambodian children and adults with uncomplicated falciparum malaria Clin Infect Dis 2002 35 1469 1476 10.1086/344647 12471565

[B13] Ezzet F Van VM Nosten F Looareesuwan S White NJ Pharmacokinetics and pharmacodynamics of lumefantrine (benflumetol) in acute falciparum malaria Antimicrob Agents Chemother 2000 44 697 704 10.1128/AAC.44.3.697-704.2000 10681341 PMC89749

[B14] Trung TN Tan B Van Phuc D Song JP A randomized, controlled trial of artemisinin-piperaquine vs dihydroartemisinin-piperaquine phosphate in treatment of falciparum malaria Chin J Integr Med 2009 15 189 192 10.1007/s11655-009-0189-6 19568711

[B15] Krudsood S Tangpukdee N Thanchatwet V Wilairatana P Srivilairit S Pothipak N Jianping S Guoqiao L Brittenham GM Looareesuwan S Dose ranging studies of new artemisinin-piperaquine fixed combinations compared to standard regimens of artemisisnin combination therapies for acute uncomplicated falciparum malaria Southeast Asian J Trop Med Public Health 2007 38 971 978 18613536 PMC3114414

[B16] Woodrow CJ Haynes RK Krishna S Artemisinins Postgrad Med J 2005 81 71 78 Review 10.1136/pgmj.2004.028399 15701735 PMC1743191

[B17] World Health Organization Guidelines for the treatment of malaria -- 2nd edition 2010 Geneva: WHO 19 21 25473692

[B18] World Health Organization Assessment and monitoring of antimalarial drug efficacy for the treatment of uncompcicated falciparum malaria 2003 Geneva: WHO

[B19] Denis MB Tsuyuoka R Lim P Lindegardh N Yi P Top SN Socheat D Fandeur T Annerberg A Christophel EM Ringwald P Efficacy of artemether-lumefantrine for the treatment of uncomplicated falciparum malaria in northwest Cambodia Trop Med Int Health 2006 11 1800 1807 10.1111/j.1365-3156.2006.01739.x 17176344

[B20] Djimdé A Lefèvre G Understanding the pharmacokinetics of Coartem Malar J 2009 Suppl 1 S4 Review 10.1186/1475-2875-8-S1-S4 PMC2760239 19818171

[B21] White NJ van Vugt M Ezzet F Clinical pharmacokinetics andpharmacodynamics and pharmacodynamics of artemetherlumefantrine Clin Pharmacokinet 1999 37 105 125 10.2165/00003088-199937020-00002 10496300

[B22] Tan B Song JP He YQ Guo WZ Guo XB Li GQ Tolerance of oral artemisinin piperaquine tablets in Chinese healthy volunteers Chin J Clin Pharmacol 2009 25 405 407

[B23] Karema C Fanello CI van Overmeir C van Geertruyden JP van Doren W Ngamije D D'Alessandro U Safety and efficacy of dihydroartemisinin/piperaquine (DHP) for the treatment of uncomplicated *Plasmodium falciparum *malaria in Rwandan children Trans R Soc Trop Med Hyg 2006 100 1105 1011 10.1016/j.trstmh.2006.01.001 16766006

[B24] Tangpukdee N Krudsood S Thanachartwet W Chalermrut K Pengruksa C Srivilairit S Silachamroon U Wilairatana P Phongtananant S Kano S Looareesuwan S An open randomized clinical trial of Artekin(DHP) vs artesunate-mefloquine in the treatment of acute uncomplicated falciparum malaria Southeast Asian J Trop Med Public Health 2005 36 1085 1091 16438129

[B25] Denis MB Davis TM Hewitt S Incardona S Nimol K Fandeur T Poravuth Y Lim C Socheat D Efficacy and safety of dihydroartemisinin-piperaquine (DHP) in Cambodian children and adults with uncomplicated falciparum malaria Clin Infect Dis 2002 35 1469 1476 10.1086/344647 12471565

[B26] Jinrong W Xinggang W Zijian L Yan L Malaria prevention survey for administration dihydroartemisinin piperaquine by officers and soldiers stationed in Sudan Prac J Med & Pharm 2009 26 36 37

[B27] Hung TY Davis TM Ilett KF Karunajeewa H Hewitt S Denis MB Lim C Socheat D Population pharmacokinetics of piperaquine in adults and children with uncomplicated falciparum or vivax malaria Br J Clin Pharmacol 2004 57 253 262 14998421 10.1046/j.1365-2125.2003.02004.x PMC1884452

[B28] Jinbo G Zijian F Huazhong L Canjun Zh Jun G Gang Zhu Fanghong Y Dongshan Zhu Analysis of side effects caused by piperaquine phosphate for malaria prophylaxis in population China Trop Med 2008 8 541 543

[B29] Brockman A Paul RE Anderson TJ Hackford I Phaiphun L Looareesuwan S Nosten F Day KP Application of genetic markers to the identification of recrudescent *Plasmodium falciparum *infections on the northwestern border of Thailand Am J Trop Med Hyg 1999 60 14 21 9988316 10.4269/ajtmh.1999.60.14

