# Supplementary material for: Randomized trials of artemisinin-piperaquine, dihydroartemisinin-piperaquine phosphate and artemether-lumefantrine for the treatment of multi-drug resistant falciparum malaria in Cambodia-Thailand border area
Source: Malar J. 2011 Aug 10;10:231. doi: 10.1186/1475-2875-10-231 (PMC3169515; doi:10.1186/1475-2875-10-231)
Supplement: Additional file 1 — Table S1 - Dosing schedules for artemisinin-piperaquine (AP), dihydroartemisinin-piperaquine phosphate (DHP) and artemether-lumefantrine (AL). *It is better to take this drug together with milk and food. [file 1475-2875-10-231-S1.DOC]

**Additional file 1, Table S1 - Dosing schedules for artemisinin-piperaquine (AP), dihydroartemisinin-piperaquine phosphate (DHP) and artemether-lumefantrine (AL)**

| **AP Tablets**  **One dose, twice daily for 1 day** | | | | **DHP Tablets**  **One dose, twice daily for 2 days** | | | | | | **AL *Tablets**  **One dose, twice daily for 3 days** | | | | | | | |
| --- | --- | --- | --- | --- | --- | --- | --- | --- | --- | --- | --- | --- | --- | --- | --- | --- | --- |
| **Ages**  **（years）** | **Dosage (tablets)** | | | **Ages**  **（years）** | **Dosage (tablets)** | | | | | **Weight**  **（kg）** | **Dosage (tablets)** | | | | | | |
|  | **0h** | **24h** | **Total dosage (tablets)** |  | **0h** | **8h** | **24h** | **32h** | **Total dosage(tablets)** |  | **D0** | | **D1** | | **D2** | | **Total dosage(tablets)** |
|  |  |  |  |  |  |  |  |  |  |  | **0h** | **8h** | **24h** | **32h** | **48h** | **56h** |  |
| ≥16 | 2 | 2 | 4 | ≥16 | 2 | 2 | 2 | 2 | 8 | ≥35 | 4 | 4 | 4 | 4 | 4 | 4 | 24 |
| 11-15 | 1.5 | 1.5 | 3 | 11-15 | 1.5 | 1.5 | 1.5 | 1.5 | 6 | 25-34 | 3 | 3 | 3 | 3 | 3 | 3 | 18 |
| 7-10 | 1 | 1 | 2 | 7-10 | 1 | 1 | 1 | 1 | 4 | 15-24 | 2 | 2 | 2 | 2 | 2 | 2 | 12 |
|  |  |  |  |  |  |  |  |  |  | 10-14 | 1 | 1 | 1 | 1 | 1 | 1 | 6 |

(*****It is better to take this drug together with milk and food)
